# Competition between cheatgrass and bluebunch wheatgrass is altered by temperature, resource availability, and atmospheric CO_2_ concentration

**DOI:** 10.1007/s00442-017-4046-6

**Published:** 2017-12-22

**Authors:** Christian D. Larson, Erik A. Lehnhoff, Chance Noffsinger, Lisa J. Rew

**Affiliations:** 1 Weed and Invasive Plant Ecology and Management Group, Land Resources and Environmental Science Department, Montana State University, Bozeman, MT 59717 USA; 2 Entomology, Plant Pathology and Weed Science, New Mexico State University, Las Cruces, NM 88003 USA

**Keywords:** *Bromus tectorum*, Climate change, Plant invasion, *Pseudoroegneria spicata*, Replacement series design

## Abstract

Global change drivers (elevated atmospheric CO_2_, rising surface temperatures, and changes in resource availability) have significant consequences for global plant communities. In the northern sagebrush steppe of North America, the invasive annual grass *Bromus tectorum* (cheatgrass) is expected to benefit from projected warmer and drier conditions, as well as increased CO_2_ and nutrient availability. In growth chambers, we addressed this expectation using two replacement series experiments designed to test competition between *B. tectorum* and the native perennial bunchgrass *Pseudoroegneria spicata*. In the first experiment, we tested the effects of elevated temperature, decreased water and increased nutrient availability, on competition between the two species. In the second, we tested the effects of elevated atmospheric CO_2_ and decreased water availability on the competitive dynamic. In both experiments, under all conditions, *P. spicata* suppressed *B. tectorum*, though, in experiment one, warmer and drier conditions and elevated nutrient availability increased *B. tectorum*’s competitiveness. In experiment two, when grown in monoculture, both species responded positively to elevated CO_2_. However, when grown in competition, elevated CO_2_ increased *P. spicata*’s suppressive effect, and the combination of dry soil conditions and elevated CO_2_ enhanced this effect. Our findings demonstrate that *B. tectorum* competitiveness with *P. spicata* responds differently to global change drivers; thus, future conditions are unlikely to facilitate *B. tectorum* invasion into established *P. spicata* communities of the northern sagebrush steppe. However, disturbance (e.g., fire) to these communities, and the associated increase in soil nutrients, elevates the risk of *B. tectorum* invasion.

**Electronic supplementary material:**

The online version of this article (10.1007/s00442-017-4046-6) contains supplementary material, which is available to authorized users.

## Introduction

Atmospheric CO_2_ concentrations have increased at an unprecedented rate since the beginning of the industrial era (IPCC 2013). It is projected that the 2015–2016 atmospheric CO_2_ concentration growth rate will be highest on record and concentrations will surpass and remain above 400 ppm for the entire year (Betts et al. 2016). The increase in atmospheric CO_2_ has altered many components of the Earth’s climate, including surface temperatures and precipitation patterns (IPCC 2013). As plant growth is directly affected by temperature, precipitation (Woodward and Williams 1987) and atmospheric CO_2_ concentrations (Bazzaz 1990), these changes have consequences for global plant communities (Shaver et al. 2000; Cramer et al. 2001; Walther et al. 2002; Parmesan and Yohe 2003; Chen et al. 2011).

Elevated atmospheric CO_2_ has long been used to stimulate plant production; however, plant attributes shape individual and group responses (Bazzaz 1990). Elevated CO_2_ favors species with intrinsically higher growth rates because they have a higher maximum rate of photosynthesis and can more fully utilize the elevated resource level (Poorter and Navas 2003). Non-native invasive plant species often have high relative growth rates; therefore, it is expected they will benefit from elevated CO_2_ (Dukes and Mooney 1999; Weltzin et al. 2003; Moore 2004; Sorte et al. 2013). Consistent with this expectation, studies have found that non-native species demonstrate greater responses to elevated CO_2_ than native species (Ziska and George 2004), and elevated CO_2_ makes them more competitive with native species when grown together (Ziska and George 2004; Manea and Leishman 2011). The presence, density, and identity of competitors influence non-native responses to elevated CO_2_ (Bazzaz et al. 1992; Wayne et al. 1999; Manea and Leishman 2011). For example, the non-native species *Centaurea solstitialis* and *Chenopodium album* responded positively to elevated CO_2_ concentrations when grown in monoculture, but failed to respond significantly when grown in competitive settings (Taylor and Potvin 1997; Dukes 2002). Resource levels also affect plant responses to elevated CO_2_ (Bazzaz 1990). For example, by reducing stomatal density and conductance and increasing plant water use efficiency (Bazzaz 1990; Polley 1997; Morgan et al. 2004), elevated CO_2_ can mitigate the negative effects of warming and drying on plant growth (Dermody et al. 2007). Conversely, in a California grassland community, the invasive annual grass *Bromus rubens* responded positively to elevated CO_2_ during wet years and negatively in dry years (Smith et al. 2014). Dukes (2000) concluded that non-native plant responses to elevated CO_2_ are context dependent, influenced by ecosystem attributes, especially water availability and species identity.

Global vegetation models and recent studies predict changes to both temperature and precipitation regimes will significantly affect plant communities (Cramer et al. 2001; Lenoir and Svenning 2015), including the spread and success of non-native invasive plant species (Milchunas and Lauenroth 1995; Weltzin et al. 2003; Everard et al. 2010; Dukes et al. 2011; Hoeppner and Dukes 2012; Wu et al. 2016). Results from both observational and experimental studies are consistent with this expectation. For example, since 1900, frost-free days in southern Switzerland have decreased while exotic species richness has increased (Walther et al. 2002). Likewise, an observed increase in minimum temperatures over a 23-year period at a Colorado shortgrass-steppe site were associated with reduced net primary production of the dominant grass species and increased exotic forb density (Alward et al. 1999). Similarly, drought is believed to be a significant contributor to the replacement of a California perennial bunchgrass ecosystem by an invasive annual grass ecosystem (Suttle and Thomsen 2007; Everard et al. 2010). Furthermore, a study in a Colorado shortgrass-steppe community found that experimentally elevated winter precipitation and decreased summer precipitation positively affected non-native invasive grass abundance and negatively affected native grass cover (Prevéy and Seastedt 2014). A recent study using a *de Wit* replacement series design with a native and non-native species found warming reduced native biomass and relative yield, especially at higher densities of the non-native species, and increased non-native biomass (Wu et al. 2016).

Recent bioclimatic envelope models indicate that the spatial extent of the invasive annual grass *Bromus tectorum* (cheatgrass) and its ecosystem dominance is likely to shift under future climate conditions (Bradley 2009; Bradley et al. 2016). *Bromus tectorum* is common throughout the western USA and has formed monoculture communities in the Columbia Basin, Great Basin, and Colorado Plateau regions (Mack 1981; Knapp 1996). Disturbance (fire and grazing) of native perennial communities facilitated its early establishment and subsequent invasion (Mack 1981; D’Antonio and Vitousek 1992). In western USA, wildfire frequency is correlated with warmer and drier conditions and is predicted to increase under future climate scenarios (Westerling et al. 2006, 2011). Fire increases soil nutrient levels (Hobbs and Huenneke 1992 and references therein; Blank et al. 2007), which is associated with increased *B. tectorum* growth and believed to contribute to *B. tectorum’s* success (D’Antonio and Vitousek 1992; Vasquez et al. 2008; He et al. 2011; Orloff et al. 2013). The increase in fire frequency and the resulting change in soil nutrients create conditions which may facilitate the spread of *B. tectorum* (Chambers and Pellant 2008).

While elevated nutrient levels may contribute to *B. tectorum’s* success, the presence of intact native perennial grass communities is the most important biotic factor limiting the spatial extent of the invasion (Brummer et al. 2016). Unfortunately, native community resilience to disturbance and resistance to *B. tectorum* invasion decreases along a climate gradient, being high in cool and moist systems and low in warm and dry systems (Chambers et al. 2007, 2014; Dodson and Root 2015). Furthermore, observational and experimental studies throughout the western USA have demonstrated that *B. tectorum* growth, cover, and abundance are associated with and respond positively to elevated temperatures and precipitation changes (Chambers et al. 2007; Compagnoni and Adler 2014a, b; Prevéy and Seastedt 2015; Blumenthal et al. 2016; Brummer et al. 2016). Similarly, single factor CO_2_ monoculture studies indicate that *B. tectorum* responds positively to elevated CO_2_ concentrations (Smith et al. 1987; Poorter 1993; Ziska et al. 2005). However, these results may not conclusively represent how *B. tectorum* will respond to future CO_2_ levels because *B. tectorum* has responded neutrally to elevated CO_2_ concentrations when evaluated in a community setting (Blumenthal et al. 2016).

*Bromus tectorum* growth and invasive success is associated with temperature, soil moisture, available nutrients, and competition with native perennial grasses; however, no research has studied the combination of all these factors in a controlled setting. Therefore, the first goal of our study was to determine if the competitive dynamic between *B. tectorum* and a recently established native perennial bunchgrass, *Pseudoroegneria spicata*, is responsive to changes in these experimental treatments. *Pseudoroegneria spicata* (bluebunch wheatgrass) is a desirable native perennial bunchgrass, common throughout western USA sagebrush–grassland communities and, when undisturbed, *P. spicata* and native grass communities limit *B. tectorum* growth and its landscape presence (Orloff et al. 2013; Brummer et al. 2016). Given the general paucity of literature on how *B. tectorum* responds to elevated CO_2_ concentrations (three monoculture studies and one community study), our second goal was to determine if competition between *B. tectorum* and recently established *P. spicata* individuals is responsive to elevated CO_2_ concentrations and if decreased water availability impacts this response. We hypothesized that in both experiments, increasing proportional density of recently established *P. spicata* individuals would have the greatest limiting effect on the establishing *B. tectorum* individuals. Secondly, we hypothesized that decreased water availability and increased temperature would favor *B. tectorum*, and that increased nutrient availability would further heighten *B. tectorum* competitiveness. Finally, we hypothesized that elevated CO_2_ concentrations and the combination of elevated CO_2_ and decreased soil water availability would increase *B. tectorum’s* competitiveness with established *P. spicata* individuals.

## Methods

### Experimental design

*Experiment 1: decreased water, increased temperature, and increased nutrient availability* This experiment was a full factorial across two water treatments, two temperature treatments, and two nutrient treatments (Online Resource 1). The treatments were replicated twice in five density combinations of *B. tectorum* seedlings and recently established *P. spicata* individuals and we performed two trials, providing four replicates of each density–treatment combination. Both trials were performed in growth chambers at the Plant Growth Center, Montana State University (MSU), Bozeman, MT (April–June and July–September, 2014).

Our temperature and water treatments were designed to represent the Southwest Montana climate in spring when plants are actively growing. Southwest Montana has cold, dry winters (November–March) and warm, dry summers (July–August) and receives a large percentage of its precipitation (46%) in the spring (April–June). For the temperature treatment, we used the mean maximum temperature and day length in June: the low temperature chamber [Temp. (−)] was 23.3 °C for the daylight period (14 h of 100 micromoles of PAR) and 6 °C for the night period (10 h); the elevated temperature chamber [Temp. (+)] was two degrees higher for both the day and night periods (25.3 and 8 °C, respectively). To control for chamber effect, growth chamber temperatures were switched every 2 weeks and the plants were moved. The water treatment was designed to simulate ambient June precipitation (77 mm) [Water (+)] and a reduction of this amount by 50% [Water (−)]. For the elevated nutrient treatment (NPK +), 173, 75.50, and 17.30 mg of slow release N, P, and K, was added, respectively.

The total target density for each pot was 50 plants pot^−1^ (988 plants m^−2^). The five density combinations of *B. tectorum* and *P. spicata* individuals were: 50:0, 37:13, 25:25, 13:37, 0:50 plants pot^−1^ (representing 988, 731, 494, 256 and 0 *B. tectorum* plants m^−2^, respectively). These densities were based on *B. tectorum* densities found locally at the Montana State University (MSU) Red Bluff Research Station near Norris, MT. Seeds were sown into circular pots (25.4 cm diameter) filled with equal parts of loam, sand, and organic matter. The soil was aerated and steam pasteurized at 70 °C for 60 min. Seeds were randomly sown within a grid with 2 cm spacing and, to account for edge effects, no seeds were sown closer than 4 cm to the sides of the pot. To simulate *B. tectorum* invasion of a recently established *P. spicata* community, the *P. spicata* seeds were sown 1 month before *B. tectorum*. The *P. spicata* seeds were the ‘Goldar’ variety obtained from the USDA Natural Resources Conservation Service, Aberdeen Plant Materials Center (Aberdeen, ID). The *B. tectorum* seeds were hand collected at the MSU Red Bluff Research Station. After the *B. tectorum* seeds were sown into the established *P. spicata*, the pots were watered evenly to facilitate germination and moved immediately to the temperature-controlled chambers, where the water and nutrient treatments were subsequently implemented. As an annual, *B. tectorum* establishes readily from seed into more established bunch grass communities; therefore, we designed the experiment so that the treatments affected *B. tectorum* germination. At the termination of the experiment, the height of ten randomly selected individuals (5 of each species in mixed pots) was recorded, and the total aboveground biomass for each pot was clipped, dried, and weighed, by species. After *B. tectorum* was sown, trials 1 and 2 were conducted for 70 and 67 days, respectively.

*Experiment 2: elevated atmospheric CO*_*2*_ *concentration and decreased water availability at an increased temperature* Under the same temperature and daylight conditions as the high temperature treatment of the previous experiment (25.3 and 8 °C, 14 h days and 10 h nights), this experiment was full factorial across two atmospheric CO_2_ concentrations and two water treatment levels. CO_2_ concentrations were: ambient [CO_2_ (−); 400 ppm] and elevated [CO_2_ (+); 800 ppm]. Similar to the previous experiment, the water treatment represented average June precipitation [Water (+)] and a 50% reduction [Water (−)]. In this experiment, we used the same soil, seed, and seeding methods as we did in the first experiment. This experiment also utilized a replacement series design with five different density combinations of *B. tectorum* individuals and established *P. spicata* individuals. However, this experiment utilized smaller square pots (11 cm × 11 cm) and the total target density for each pot was 12 plants pot^−1^ (1000 plants m^−2^). Thus, the five density combinations of *B. tectorum* individuals and *P. spicata* individuals were: 12:0, 9:3, 6:6, 3:9, 0:12 plants pot^−1^ (1000, 750, 500, 250, 0 *B. tectorum* plants m^−2^, respectively). There were six replicates of each density (5-level), CO_2_ (2-level), and water (2-level) treatment, resulting in a total of 120 pots per trial. Like the first experiment, we performed two trials of this experiment (January–April 2015 and May–August 2015). The trials were run for 69 and 54 days after *B. tectorum* was sown in the first and second trial, respectively. Upon the termination of the experiment, the final height was taken from all individuals in the pot and the aboveground biomass was clipped, dried, and weighed.

### Statistical analysis

To evaluate the competition effects between *B. tectorum* (*B*) and *P. spicata* (*P*) under the different treatment conditions, we calculated relative yield (RY) using the proportion of the species in mixture (*P*), mean species biomass of the species in mixture (mix), and mean species biomass in monoculture (mon), using the following formulae (Cousens and Neill 1993):

$${\text{RY}}_{\text{B}} = P_{\text{B}} \times \left( {B_{\text{mix}} /B_{\text{mon}} } \right),$$

$${\text{RY}}_{\text{P}} = P_{\text{P}} \times \left( {P_{\text{mix}} /P_{\text{mon}} } \right).$$

Relative yield indicates resource demands of the separate species and the shape of the curve indicates species interference (Burnett and Mealor 2015). If the species compete equally against one another, the RY should equal the expected proportion of the plants in the pot (Burnett and Mealor 2015). We also derived total relative yield (RYT), the sum of each species’ relative yield in each pot (Weigelt and Jolliffe 2003). RYT below 1 likely indicates competitive interference (Burnett and Mealor 2015). RYT is often interpreted using diagrams and can reveal interference (in)equalities and can indicate the direction of the imbalances (Weigelt and Jolliffe 2003). Both RY and RYT use a constant derived from an unknown amount of intraspecific competition (Weigelt and Jolliffe 2003); therefore, we also calculated the specific proportion of the total biomass for each pot. Finally, we analyzed the effects of competition and the treatments on the mean final height and aboveground biomass of each species within each pot.

We analyzed the effects of competition and the treatments using linear mixed-effects models. All models were fit with the experimental treatments and the proportion of *P. spicata* in each pot as fixed effects and trial as a random effect. To satisfy model assumptions of normality and heteroscedasticity, the following data were transformed for the first experiment: *B. tectorum* relative yield and biomass were square root transformed, *P. spicata* relative yield was logit transformed, and the species proportion of the total biomass was logit transformed. In the second experiment, the following data were transformed: *B. tectorum* height and biomass were both log transformed, *B. tectorum* relative yield was logit transformed, *P. spicata* biomass and relative yield were square root transformed, RYT was also square root transformed, and the species proportion of the total biomass was logit transformed. The initial models included interactions and were reduced to the most parsimonious model with experimental treatments still included. The target proportions of each species were not always achieved; therefore, we calculated and used the actual proportion of *P. spicata* in each pot, creating a continuous variable that was used in the analysis and the graphics. The proportion of *P. spicata* within each pot was used as an explanatory variable for both *P. spicata* and *B. tectorum*, because it was established prior to *B. tectorum* and it better explained the variation the competitive pots than did the proportion of *B. tectorum*. The analyses were conducted using the statistical program R (version 3.2.2, R Development Core Team 2015). Linear mixed-effects models were constructed using the lme4 package (Bates et al. 2011) and the lmerTest package (Kuznetsova et al. 2014). Significant relationships between the treatment effects and the response variables were calculated at the *P* < 0.05 level from T-statistics based on Satterthwaite’s approximations of degrees of freedom for mixed-effects models (Kuznetsova et al. 2014).

## Results

### Effects of competition, decreased water, elevated temperature, and increased nutrient availability

**Fig. 1:**
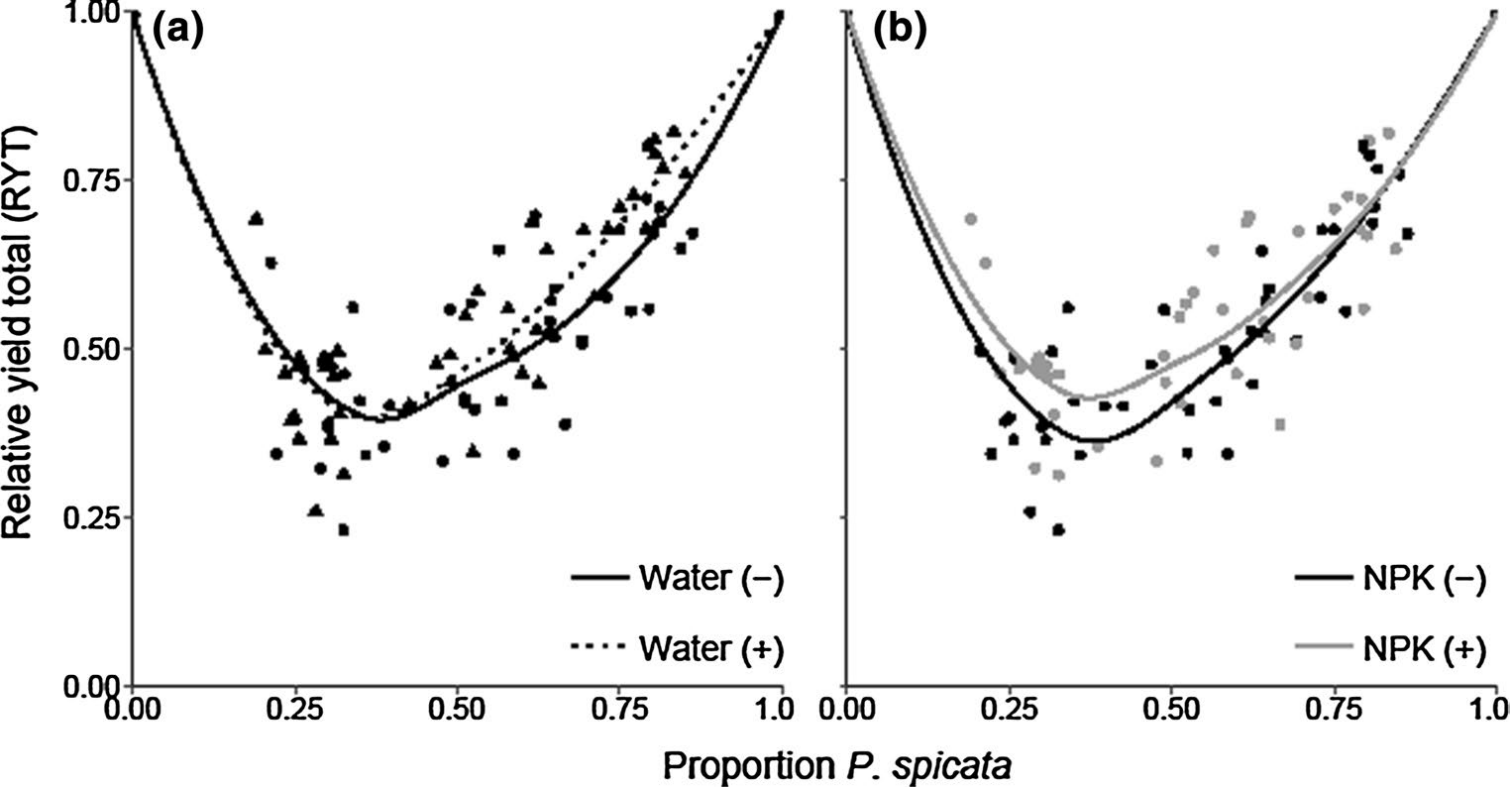
The relative yield total (RYT) for **a** water treatments: ambient (dotted line, solid triangles) and decreased (solid line, solid circles); **b** nutrient treatments: ambient (black line, solid circles) and elevated (gray line, solid triangles). A mixed-effects model demonstrated that RYT responded negatively to water availability (*n* = 86, *P* = 0.030), but positively to elevated nutrient availability (*n* = 86, *P* = 0.027) and pot proportion of *Pseudoroegneria spicata* (*n* = 86, *P* < 0.001)

*Bromus tectorum* relative yield responded positively to elevated temperature and increased nutrients (*P* = 0.038 and *P* = 0.003, respectively), demonstrated no response to water availability (*P* = 0.165), and negatively to the pot proportion of *P. spicata* (*P* < 0.001; Table 1). The decreased water and elevated temperature treatments negatively affected *P. spicata* relative yield (*P* < 0.001 and *P* = 0.029, respectively). However, there was an interaction between these variables (*P* = 0.036), indicating that the mean *P. spicata* relative yield in warm and dry conditions was greater than the mean in ambient and dry conditions. *Pseudoroegneria spicata* relative yield was unresponsive to the nutrient treatment (*P* = 0.702) and responded positively to the proportion of *P. spicata* within the pot (*P* < 0.001; Table 1). RYT was significantly affected by proportion of *P. spicata* and was below 1, indicating interference between the two species (*P* < 0.001; Fig. 1). The decreased water treatment had a negative effect on RYT, indicating increased competitive interference between the two species (*P* = 0.030; Fig. 1a). RYT responded positively to the increased nutrient treatment, indicating that the nutrient treatment decreased competitive interference (*P* = 0.027; Fig. 1b; Table 2).

**Table 1:** Results of the linear mixed-effects models assessing the effects of experimental treatments on *B. tectorum* and *P. spicata* aboveground biomass (g), height (cm), and relative yield (RY), for the first experiment

Fixed effects							Random effects	
Response	Predictor	Est.	SE	*df*	*t* value	*P* (>)	Variance	
							Trial	Residual
*B. tectorum*								
Biomass (g)	Intercept	1.72	0.15	1.31	11.39	0.029	0.038 ± 0.20	0.12 ± 0.34
	Water (−)	− 0.18	0.06	111.00	− 2.88	0.005		
	Temp (+)	− 0.04	0.06	111.03	− 0.67	0.505		
	NPK (+)	0.26	0.06	111.01	4.14	<0.001		
	*P. spicata*	− 10.79	0.48	111.05	− 22.49	<0.001		
	Water (−) × *P. spicata*	1.96	0.68	111.00	2.86	0.005		
Height (cm)	Intercept	19.85	1.54	1.20	12.86	0.031	4.18 ± 2.05	8.22 ± 2.87
	Water (−)	− 3.50	0.56	100.00	− 6.27	<0.001		
	Temp (+)	− 0.01	0.56	100.02	− 0.02	0.982		
	NPK (+)	2.01	0.56	100.01	3.60	<0.001		
	*P. spicata*	− 40.24	2.96	100.44	− 13.58	<0.001		
Relative yield (RY)	Intercept	− 3.30	0.32	1.30	− 10.20	0.033	0.17 ± 0.42	0.40 ± 0.63
	Water (−)	− 0.19	0.14	79.00	1.40	0.165		
	Temp (+)	0.29	0.14	79.04	2.11	0.038		
	NPK (+)	0.43	0.14	79.02	3.12	0.003		
	*P. spicata*	− 13.90	0.65	79.36	− 21.50	<0.001		
	*P. spicata* ^2^	− 1.29	0.64	79.16	− 2.02	0.047		
*P. spicata*								
Biomass (g)	Intercept	8.31	1.59	1.06	5.23	0.109	4.85 ± 2.20	2.36 ± 1.54
	Water (−)	− 2.50	0.4	111.00	− 6.30	<0.001		
	Temp (+)	− 1.22	0.28	111.01	− 4.29	<0.001		
	NPK (+)	2.16	0.40	111.01	5.42	<0.001		
	*P. spicata*	15.39	1.55	111.03	9.92	<0.001		
	Water (−) × NPK (+)	− 1.92	0.57	111.00	− 3.38	0.001		
Height (cm)	Intercept	34.84	0.63	102.00	55.15	<0.001	0.00 ± 0.00	11.37 ± 3.37
	Water (−)	− 5.99	0.65	102.00	− 9.18	<0.001		
	Temp (+)	− 2.21	0.65	102.00	− 3.39	<0.001		
	NPK (+)	1.10	0.65	102.00	1.69	0.094		
	*P. spicata*	− 13.48	3.37	102.00	− 4.00	<0.001		
Relative yield (RY)	Intercept	− 0.22	0.09	79.00	− 2.41	0.018	0.00 ± 0.00	0.15 ± 0.39
	Water (−)	− 0.43	0.12	79.00	− 3.71	<0.001		
	Temp (+)	− 0.26	0.12	79.00	− 2.22	0.029		
	NPK (+)	− 0.03	0.08	79.00	− 0.38	0.702		
	*P. spicata*	9.73	0.39	79.00	25.15	<0.001		
	*P. spicata* ^2^	− 0.82	0.39	79.00	− 2.11	0.038		
	Water (−) × Temp (+)	0.36	0.17	79.00	2.13	0.036		

Experimental treatments were: competition with *Pseudoroegneria spicata*, decreased water availability, water (−), increased temperature, temp. (+), and increased nutrient availability, NPK (+). Response variables were assessed in pots with 0.2 m^−2^ area. Random effects are the mean trial and residual variance and the associated standard deviation

**Table 2:** Results of the best linear mixed-effects models assessing the impact of the experimental treatments on *Bromus tectorum* and *Pseudoroegneria spicata* proportion of the total pot biomass (prop. biomass) and relative yield total (RYT) for both experiments

Fixed effects							Random effects	
Response	Predictor	Est.	SE	*df*	*t* value	*P* (>)	Variance	
							Trial	Residual
Experiment 1								
Prop. biomass	Intercept	− 2.18	0.60	1.09	− 3.61	0.155	0.68 ± 0.83	0.49 ± 0.70
	Water (−)	0.40	0.15	80.00	2.68	0.009		
	Temp (+)	0.28	0.15	80.01	1.83	0.071		
	NPK (+)	0.46	0.15	80.01	3.02	0.003		
	*P. spicata*	− 10.23	0.72	80.12	− 14.30	<0.001		
Relative yield total (RYT)	Intercept	0.51	0.02	2.89	22.24	<0.001	<0.001 ± 0.02	0.007 ± 0.08
	Water (−)	− 0.04	0.02	79.00	− 2.21	0.030		
	Temp (+)	0.03	0.02	79.20	1.74	0.086		
	NPK (+)	0.04	0.02	79.08	2.26	0.027		
	*P. spicata*	0.96	0.08	80.00	11.43	<0.001		
	*P. spicata* ^*2*^	0.46	0.08	79.61	5.59	<0.001		
Experiment 2								
Prop. biomass	Intercept	− 1.23	0.32	1.46	− 3.87	0.098	0.15 ± 0.39	1.15 ± 1.07
	CO_2_ (+)	− 1.66	0.19	131.00	− 8.82	<0.001		
	Water (−)	− 0.15	0.18	131.00	− 0.80	0.428		
	*P. spicata*	− 11.29	1.74	131.61	− 6.50	<0.001		
	*P. spicata* ^*2*^	5.69	1.84	131.06	− 3.10	0.002		
	*P. spicata* × CO_2_	3.81	2.42	131.33	1.57	0.118		
	*P. spicata* ^2^ × CO_2_	− 6.89	2.38	131.12	− 2.89	0.004		
Relative yield total (RYT)	Intercept	0.68	0.05	1.19	13.58	0.030	0.004 ± 0.07	0.01 ± 0.12
	CO_2_ (+)	− 0.02	0.02	134.00	− 1.13	0.259		
	Water (−)	− 0.01	0.02	134.00	− 0.57	0.571		
	*P. spicata*	0.98	0.12	134.14	7.94	<0.001		

The experimental treatments assessed for experiment one: *P. spicata* pot density, decreased water availability, water (−), increased temperature, temp. (+), and increased nutrient availability, NPK (+). The experimental treatments assessed for experiment two: *P. spicata* pot density, decreased water availability, water (−), and elevated atmospheric CO_2_ concentration, CO_2_ (+). The random effects are the mean trial and residual variance and the associated standard deviation

**Fig. 2:**
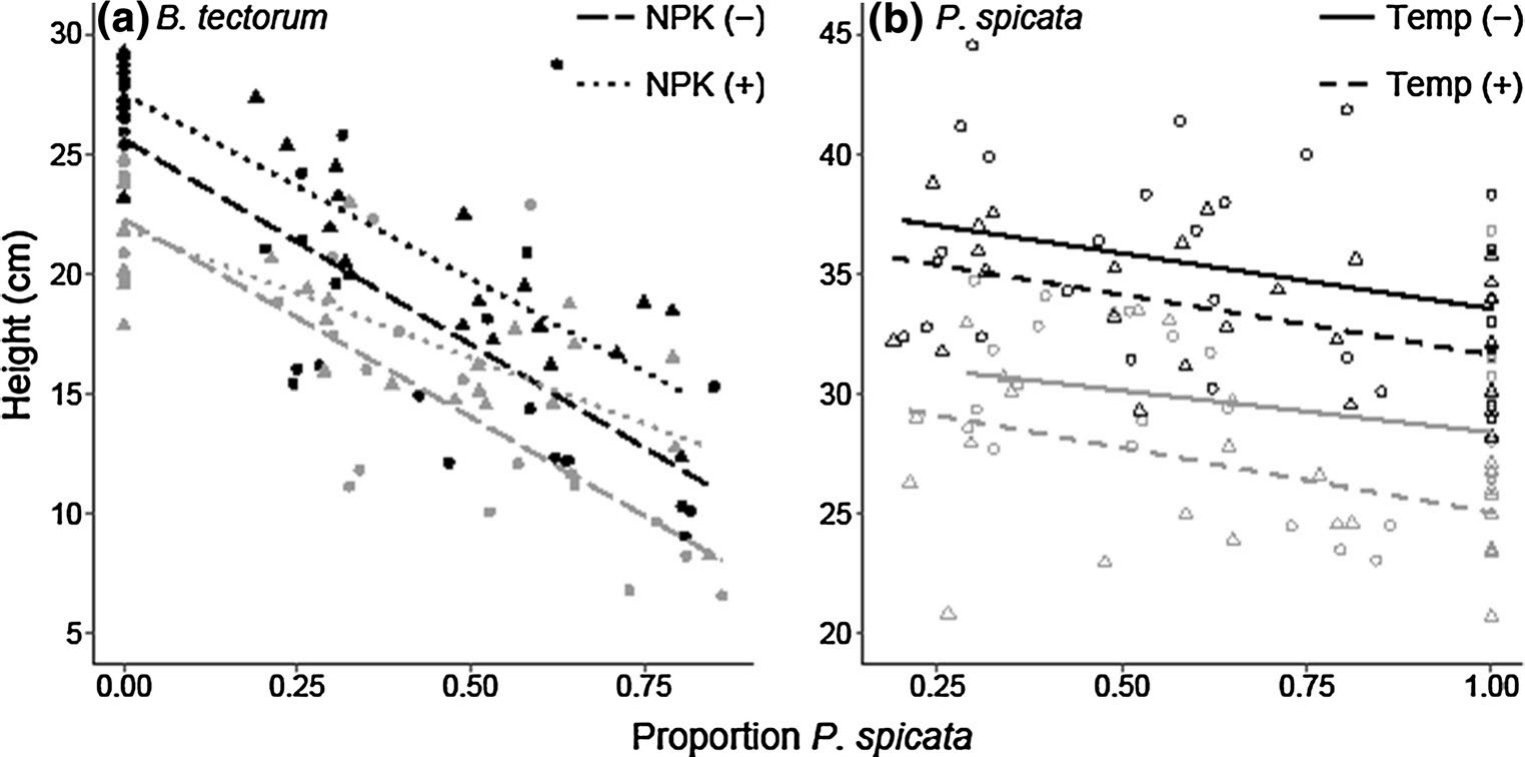
Height response under ambient and decreased water (black and gray, respectfully) for **a** *Bromus tectorum* in ambient (long dashed line, solid circles) and elevated (dotted line, solid triangles) nutrients and **b** *Pseudoroegneria spicata* in ambient (solid line, open circle) and elevated (short dash line, open triangles) temperature. Mixed-effects models demonstrated: **a** *B. tectorum* height was negatively affected by *P. spicata* pot proportion (*n* = 106, *P* < 0.001) and decreased water (*n* = 106, *P* < 0.001), while it responded positively to increased nutrients (*n* = 64, *P* < 0.001); **b** *P. spicata* height was negatively affected by *P. spicata* pot proportion (*n* = 106, *P* < 0.001), increased temperature (*n* = 106, *P* < 0.001), and decreased water (*n* = 106, *P* < 0.001)

*Bromus tectorum* aboveground biomass responded negatively to the interspecific competition with the established *P. spicata* individuals (*P* < 0.001), and decreased water availability (*P* = 0.005), while it responded positively to nutrient addition (*P* < 0.001; Table 1). There was an interaction between the interspecific competition and water availability (*P* = 0.005; Table 1); the suppressive effect of *P. spicata* on *B. tectorum* aboveground biomass was greater at high *P. spicata* proportions in the watered treatment than in the decreased water treatment. *Bromus tectorum* height responded negatively to interspecific competition (*P* < 0.001) and decreased water availability (*P* < 0.001), while it responded positively to increased nutrient availability (*P* < 0.001; Fig. 2a; Table 1). Neither *B. tectorum* biomass nor height responded to elevated temperature (Table 1).

*Pseudoroegneria spicata* aboveground biomass responded negatively to decreased water availability (*P* < 0.001) and elevated temperature (*P* < 0.001), but responded positively to nutrient availability (*P* < 0.001) and increased *P. spicata* proportion (*P* < 0.001). There was an interaction between the water treatment and the nutrient treatment (*P* = 0.001); in the watered treatment, added nutrients had a positive effect while in the ambient water treatment added nutrients had no effect. *P. spicata* height responded negatively to decreased water availability (*P* < 0.001), increased temperature (*P* < 0.001), and when *P. spicata* proportion increased (*P* < 0.001; Fig. 2b), but did not demonstrate a response to the increased nutrient availability treatment (*P* = 0.094; Table 1).

*Bromus tectorum* proportion of the total pot biomass responded positively to decreased water availability (*P* = 0.009) and increased nutrient availability (*P* = 0.003), and there was minimal evidence that increased temperature also had a positive effect (*P* = 0.071; Table 2). The proportion of *P. spicata* within the pot negatively affected the pot biomass *B. tectorum* (*P* < 0.001; Table 2).

### Effects of competition, elevated atmospheric CO_2_, and decreased water availability under an increased temperature

**Fig. 3:**
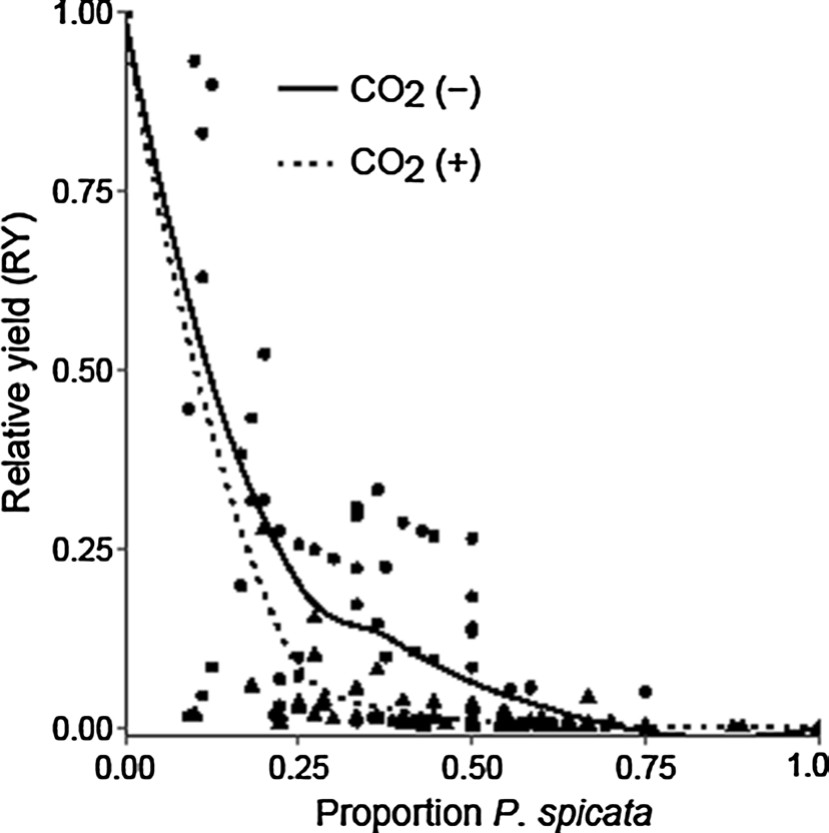
*Bromus tectorum* relative yield in the ambient (solid line, solid circles) and elevated (dotted line, solid triangles) atmospheric CO_2_ treatments. A mixed-effects model demonstrated that elevated CO_2_ (*n* = 139) and competition with *P. spicata* (*n* = 139) had negative effects on *B. tectorum* relative yield (*P* < 0.001 and *P* < 0.001, respectively)

**Fig. 4:**
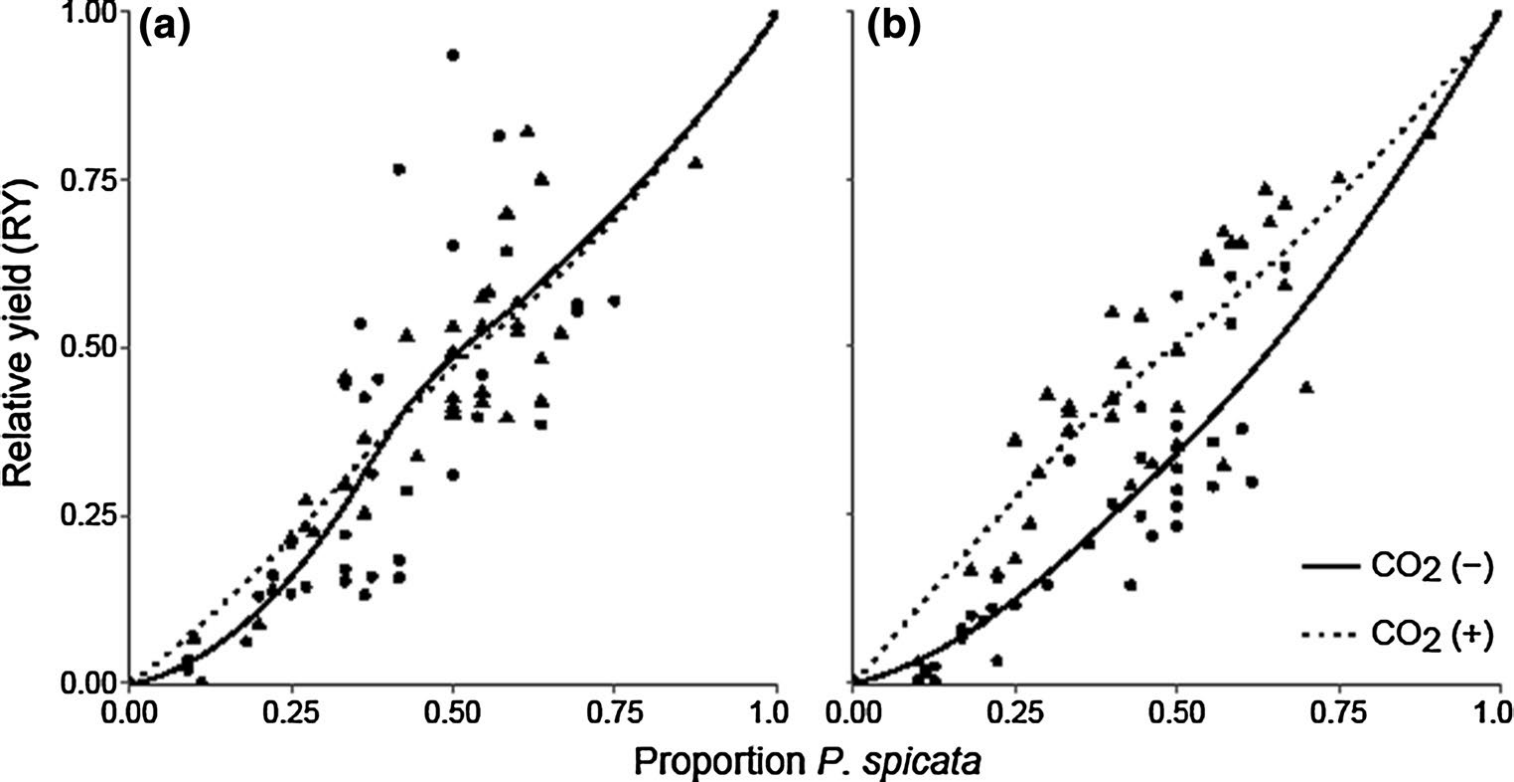
*Pseudoroegneria spicata* relative yield response in **a** ambient and **b** decreased water treatments to ambient (solid line, solid circles) and elevated (dotted line, solid triangles) atmospheric CO_2_. A mixed-effects model demonstrated that *P. spicata* pot proportion (*n* = 139, *P* < 0.001) positively affected *P. spicata* relative yield and elevated CO_2_ had no effect (*n* = 139, *P* = 0.233), while decreased water availability had a negative effect (*n* = 139, *P* = 0.001). There was a significant interaction between the CO_2_ and water treatments (*P* < 0.001): *P. spicata* relative yield demonstrated no response to elevated CO_2_ in the **a** ambient water treatment and a positive response in the **b** decreased water treatment

*Bromus tectorum* relative yield responded negatively to elevated CO_2_ and the pot proportion of *P. spicata* (*P* < 0.001 and *P* < 0.001, respectively; Fig. 3), but demonstrated no response to the decreased water treatment (*P* = 0.339; Table 3). In contrast, *P. spicata* relative yield demonstrated no response to the elevated CO_2_ treatment (*P* = 0.233), a negative response to the decreased water treatment (*P* = 0.001), and there was an interaction between the two factors (*P* < 0.001; Fig. 4; Table 3): *P. spicata* relative yield demonstrated no response to elevated CO_2_ in the ambient water treatment (Fig. 4a), but when water availability was reduced *P. spicata* relative yield responded positively to elevated CO_2_ (Fig. 4b). *Pseudoroegneria spicata* relative yield responded positively when its pot proportion increased (*P* < 0.001; Fig. 4; Table 3). Neither the elevated CO_2_ treatment nor the decreased water treatment affected the RYT (*P* = 0.259 and *P* = 0.571, respectively; Table 2), while the proportion of *P. spicata* did affect the RYT, which was below 1 indicating competitive interference between the species (*P* < 0.001; Table 2).

**Table 3:** Results of the linear mixed-effects models assessing the effects of experimental treatments on *B. tectorum* and *P. spicata* aboveground biomass (g), height (cm), and relative yield (RY), for the second experiment. Experimental treatments were: competition with *Pseudoroegneria spicata,* elevated atmospheric CO_2_, CO_2_ (+), and decreased water, water (−)

Fixed effects							Random effects	
Response	Predictor	Est.	SE	*df*	*t* value	*P* (>)	Variance	
							Trial	Residual
*B. tectorum*								
Biomass (g)	Intercept	− 0.38	0.51	1.06	− 0.74	0.587	0.49 ± 0.70	0.69 ± 0.83
	CO_2_ (+)	− 0.31	0.12	178.00	− 2.53	0.012		
	Water (−)	− 0.30	0.12	178.00	− 2.47	0.014		
	*P. spicata*	− 15.55	1.30	178.07	− 11.97	<0.001		
	*P. spicata* ^*2*^	3.74	0.12	178.07	2.60	0.010		
	*P. spicata* × CO_2_ (+)	− 7.33	1.73	178.03	− 4.24	<0.001		
Height (cm)	Intercept	2.60	0.26	1.03	9.88	0.060	0.13 ± 0.37	0.10 ± 0.31
	CO_2_ (+)	− 0.09	0.05	177.00	− 1.97	0.050		
	Water (−)	− 0.08	0.05	177.00	− 1.73	0.086		
	*P. spicata*	− 3.55	0.47	177.02	− 7.51	<0.001		
	*P. spicata* × CO_2_ (+)	− 1.96	0.64	177.01	− 3.07	0.002		
Relative yield (RY)	Intercept	− 2.98	0.96	1.03	− 3.10	0.193	1.80 ± 1.34	0.86 ± 0.92
	CO_2_ (+)	− 1.52	0.16	134.00	− 9.39	<0.001		
	Water (−)	0.15	0.16	134.00	0.96	0.339		
	*P. spicata*	− 12.65	0.96	134.02	− 13.20	<0.001		
*P. spicata*								
Biomass (g)	Intercept	1.38	0.38	1.01	3.60	0.170	0.29 ± 0.54	0.08 ± 0.28
	CO_2_ (+)	0.57	0.04	178.00	13.48	<0.001		
	Water (−)	− 0.23	0.04	178.00	− 5.62	<0.001		
	*P. spicata*	2.88	0.39	178.00	7.41	<0.001		
	*P. spicata* ^2^	− 1.87	0.39	178.01	− 4.85	<0.001		
	*P. spicata* × CO_2_ (+)	− 2.32	0.58	178.00	− 3.98	<0.001		
	*P. spicata* ^2^ × CO_2_ (+)	1.59	0.59	178.00	2.70	0.008		
Height (cm)	Intercept	24.47	2.17	1.07	11.27	0.048	8.95 ± 3.00	14.54 ± 3.81
	CO_2_ (+)	10.52	0.56	181.00	18.71	<0.001		
	Water (−)	− 2.17	0.56	181.00	− 3.89	<0.001		
	*P. spicata*	5.86	5.22	181.03	1.12	0.263		
	*P. spicata* × CO_2_ (+)	− 28.16	7.72	181.02	− 3.65	<0.001		
Relative yield (RY)	Intercept	0.57	0.02	8.62	33.80	<0.001	<0.001 ± 0.008	0.009 ± 0.09
	CO_2_ (+)	0.03	0.02	132.00	1.198	0.233		
	Water (−)	− 0.07	0.02	132.01	− 3.33	0.001		
	*P. spicata*	1.90	0.10	132.00	19.72	<0.001		
	*P. spicata* ^*2*^	− 0.51	0.09	132.45	− 5.50	<0.001		
	CO_2_ (+) × Water (−)	0.10	0.03	132.02	3.43	<0.001		

Response variables were assessed in pots with 0.1 m^−2^ area. Random effects are the mean trial and residual variance and the associated standard deviation

*Bromus tectorum* biomass and height responded negatively to both interspecific competition with *P. spicata* (*P* < 0.001 and *P* < 0.001, respectively) and the elevated CO_2_ treatment (*P* = 0.012, *P* = 0.050, respectively; Table 3). In addition, decreased water negatively affected *B. tectorum* biomass (*P* = 0.014), but did not affect its height (*P* = 0.086; Table 3). An interaction between increasing *P. spicata* pot density and the CO_2_ treatment affected both *B. tectorum* aboveground biomass and height (*P* < 0.001 and *P* = 0.002, respectively; Table 3); under elevated CO_2_, the effects of increasing *P. spicata* pot density (greater interspecific competition) were magnified.

*Pseudoroegneria spicata* biomass and height responded positively to elevated CO_2_ (*P* < 0.001 and *P* < 0.001, respectively; Table 3). However, as the proportion of *P. spicata* increased, only its biomass responded positively (*P* < 0.001; Table 3). Both *P. spicata* biomass and height responded negatively to the decreased water treatment (*P* < 0.001, *P* < 0.001, respectively; Table 3). For both response variables, there was an interaction between *P. spicata* density and CO_2_ (*P* < 0.001, *P* < 0.001, respectively; Table 3): as *P. spicata* density increased, the mean biomass in the ambient CO_2_ treatment increased at a greater rate than did the mean biomass within the elevated CO_2_ treatment; and, as *P. spicata* density increased, its mean height actually decreased in the elevated CO_2_ treatment, while its mean height in the ambient CO_2_ treatment was unaffected by the increasing density.

Elevated CO_2_ positively affected the biomass and height of both species when they were grown in monoculture. In monoculture, *B. tectorum* mean biomass in the elevated CO_2_ treatment (8.49 g) was greater than its mean biomass in ambient CO_2_ (5.09 g; *P* < 0.001, *t* statistic = 12.56, *df* = 43). Likewise, the mean height of *B. tectorum* grown in monoculture under elevated CO_2_ (25.66 cm) was taller than its mean height in ambient CO_2_ (21.34 cm; *P* < 0.001, *t* statistic = 6.19, *df* = 43). When grown in monoculture, *P. spicata* mean biomass in the elevated CO_2_ treatment (4.55 g) was greater than its mean biomass in ambient CO_2_ (2.88 g; *P* < 0.001, *t* statistic = 5.22, *df* = 43). Similarly, the mean height of *P. spicata* in the elevated CO_2_ monoculture treatment (33.12 cm) was taller than its mean height in the ambient CO_2_ monoculture treatment. (22.66 cm; *P* < 0.001, *t* statistic = 10.23, *df* = 44).

*Pseudoroegneria spicata* pot proportion negatively and positively affected the *B. tectorum* and *P. spicata* relative contributions to total pot biomass, respectively (*P* < 0.001). Likewise, the response of the proportion pot biomass to the elevated CO_2_ treatment demonstrated the same trend (*P* < 0.001; Table 2). The water treatment did not affect the species proportion of the total pot biomass (*P* = 0.428; Table 2).

## Discussion

Established native perennial grasses, including *P. spicata*, are highly competitive with *B. tectorum* (Orloff et al. 2013; Prevéy and Seastedt 2015; Larson et al. 2017) and are the most significant biotic factor limiting *B. tectorum* distribution in the sagebrush biome (Brummer et al. 2016). Consistent with the literature and as expected, in both of our experiments and under all conditions, interspecific competition with established *P. spicata* individuals was the most significant factor limiting *B. tectorum* growth.

*Bromus tectorum* biomass and fecundity can respond positively to experimental warming in competitive community settings (Zelikova et al. 2013; Compagnoni and Adler 2014a, b; Blumenthal et al. 2016). However, *B. tectorum* cover, density, and fecundity have also been found to respond negatively to experimental warming when in a competitive community setting (Larson et al. 2017). Interestingly, our results were consistent with both sets of studies: *B. tectorum* individual metrics (height and biomass) failed to respond to our elevated temperature treatment, while *B. tectorum* relative yield, which accounts for the effects of interspecific competition, responded positively to warmer temperatures. The latter response is likely due to *P. spicata’s* substantial negative response to the experimental warming and demonstrates an apparent increase in *B. tectorum* competitiveness under these conditions. As experimental warming can negatively affect bunchgrass ecosystems (Carlyle et al. 2014), and *P. spicata* has demonstrated a limited ability to adapt to altered climate conditions (Fraser et al. 2009), the *P. spicata* negative responses and *B. tectorum’s* competitiveness under warmer conditions were expected and support our first hypothesis. *Pseudoroegneria spicata* relative yield did demonstrate an interaction between temperature and water availability; however, when compared to the reference level (ambient water, ambient temperature), the effects of the interaction were still negative.

*Bromus tectorum* and *P. spicata* respond negatively to decreased water availability (Cline et al. 1977; Chambers et al. 2007; Fraser et al. 2009; Prevéy and Seastedt 2015; Larson et al. 2017). Consistent with these findings, we found that individually, both species responded negatively to the lower water treatment. However, *B. tectorum’s* shallow diffuse root structure lends it a greater ability to extract water from extremely dry soil, which increases its competitiveness with its native perennial neighbors when water is limiting (Harris 1967; Eissenstat and Caldwell 1988; Link et al. 1990). The specific proportion of the total biomass results, a metric used to evaluate the competition between the two species, demonstrated *B. tectorum’s* competitiveness in dry conditions. These results, in addition to *P. spicata*’s individual negative responses to both the warming and drying treatments, support our hypothesis that increased temperature and decreased water alter the competitive dynamics in favor of the invasive *B. tectorum*. One of the limitations of our study was that, being a growth chamber study, we were only able to decrease the quantity of soil water and could not address the importance of the seasonality of soil moisture availability (Prevéy and Seastedt 2015; Larson et al. 2017).

Studies have found that non-native invasive plant species outcompete native plants in high resource environments, while native plants are more successful in low resource areas (Davis et al. 2000; Daehler 2003; though see Maron and Marler 2008). *Bromus tectorum* is associated with areas where elevated nutrients are present (Norton et al. 2004), and it is a very strong competitor for available soil nutrients, especially nitrogen (Booth et al. 2003); thus, its successful invasion and response to fire have been tied to increased availability of soil nutrients (D’Antonio and Vitousek 1992; Vasquez et al. 2008; He et al. 2011; Orloff et al. 2013). Consistent with the literature, the addition of nutrients had positive effects on all *B. tectorum* response variables (biomass, height, and relative yield) in our first experiment. Similarly, the addition of nutrients combined with the decreased water treatment increased the *B. tectorum* proportion of the total pot biomass and increased RYT, indicating a decrease in competitive interference by *P. spicata*. There was generally a lack of response by *P. spicata* to the nutrient addition. Consistent with our hypothesis, this suggests that, while interspecific competition with the larger *P. spicata* still limited *B. tectorum*, added nutrients did increase *B. tectorum’s* competitiveness, decreased *P. spicata*’s competitive interference, and drier conditions exaggerated this effect.

Elevated CO_2_ concentrations have consistently been associated with increased growth, especially for C3 species (Bazzaz 1990; Poorter 1993; Ackerly and Bazzaz 1995; Polley 1997; Poorter and Navas 2003) including *B. tectorum* (Smith et al. 1987; Poorter 1993; Ziska et al. 2005). A mechanism through which atmospheric CO_2_ concentrations facilitate plant growth is by increasing plant water use efficiency (Bazzaz 1990); therefore, soil water relations will mediate and affect how plant communities respond to increasing CO_2_ (Bazzaz et al. 1992; Morgan et al. 2004; Smith et al. 2014). A long-term free-air carbon dioxide enrichment (FACE) plant community study (Smith et al. 2014) demonstrated the importance of this interaction for annual grasses. Smith et al. (2014) demonstrated that responses by an annual *Bromus* spp. to elevated CO_2_ were highly contingent on soil moisture. Thus, the second goal of our study was to assess the impact of elevated atmospheric CO_2_ concentrations on the competition between *P. spicata* and *B. tectorum* and to determine if these effects were responsive to a 50% reduction in water availability.

Consistent with the results of the other *B. tectorum* CO_2_-controlled setting studies (Smith et al. 1987; Ziska et al. 2005) when *B. tectorum* and *P. spicata,* were grown in monoculture, elevated CO_2_ had positive effects. However, when grown in competition, our findings were inconsistent with these findings and those of the only relevant field experiment, which found *B. tectorum* responded neutrally to elevated CO_2_ in a native Wyoming mixed prairie community (Blumenthal et al. 2016). We found that the individual metrics (height, biomass) of the established *P. spicata* responded positively to elevated CO_2_, while the same metrics of the younger *B. tectorum* plants responded negatively. Furthermore, the effective changes in the specific proportion of total pot biomass and relative yield clearly demonstrated that elevated CO_2_ provided the established *P. spicata* with an even greater competitive edge. The decreased water treatment had no effect on *B. tectorum’s* response to elevated CO_2_ and it was clear that when soil moisture was reduced, a condition which has previously been shown to favor *B. tectorum*, *P. spicata* greatly benefitted from the elevated CO_2_ concentration.

Resource availability and the presence of neighbors influence competitive and community responses to elevated CO_2_; therefore, they often differ significantly from monoculture responses (Ackerly and Bazzaz 1995; Shaw et al. 2002; Smith et al. 2014). There are two likely mechanisms underlying *B. tectorum’s* response to elevated CO_2_. First, *B. tectorum* is strongly limited by interspecific competition with established *P. spicata* (Orloff et al. 2013) and the elevated CO_2_ enhanced the size and competitive advantage, especially for light, of the established *P. spicata* individuals; thus, the effects of increased interspecific competition overwhelmed any positive effects the increased CO_2_ had on *B. tectorum*. The second mechanism potentially underlying *B. tectorum’s* response to elevated CO_2_ while in competition could be the indirect effects of the CO_2_ on available N. Elevated CO_2_ commonly reduces N availability (Luo et al. 2004), which could moderate the positive effects that elevated CO_2_ might hold for invasive species (Sorte et al. 2013; Blumenthal et al. 2016), especially those that are responsive to heightened nutrient availability, such as *B. tectorum*. Additionally, *B. tectorum’s* response could be the result of an interaction between the two mechanisms: the larger *P. spicata* used more soil nutrients, thereby causing soil nutrient limitation for the smaller, less competitive, *B. tectorum*.

*Bromus tectorum* is highly competitive with native perennial grasses when moisture is limiting (Harris 1967; Eissenstat and Caldwell 1988). However, we found in elevated CO_2_ that *P. spicata* experienced less competition with *B. tectorum* in dry conditions. One potential explanation for this result is an improvement of *P. spicata* water use efficiency under these conditions, and is a common effect that elevated CO_2_ can have on C_3_ species (Bazzaz 1990). Another potential explanation is that the larger *P. spicata* individuals had larger root systems and, thus, obtained a greater amount of the limiting resource than the smaller *B. tectorum* individuals. Such a response by one of *B. tectorum’s* perennial bunchgrass competitors could limit invasion success of *B. tectorum* under future climate conditions and CO_2_ concentrations.

The sagebrush-steppe biome in the western USA is projected to become warmer with more variable precipitation, and with more frequent wildfires (Chambers and Pellant 2008; Bradley 2009; Mote and Salathé 2010; Westerling et al. 2011); thus, it has been postulated *B. tectorum’s* range will expand (Bradley 2009; Taylor et al. 2014; Bradley et al. 2016). Furthermore, elevated atmospheric CO_2_ concentrations are expected to favor invasive species (Dukes and Mooney 1999; Weltzin et al. 2003; Ziska and George 2004; Thuiller et al. 2008), including *B. tectorum* (Smith et al. 1987; Ziska et al. 2005). Our findings demonstrate that recently established *P. spicata* significantly suppresses *B. tectorum*, especially in elevated atmospheric CO_2_. Furthermore, despite *B. tectorum* experiencing an increase in competitiveness under decreased water, increased temperature, and elevated nutrient conditions, this suppressive effect was still the most significant factor affecting *B. tectorum* growth. Being conducted in a greenhouse under controlled settings, our study has limitations; specifically, we were unable to address the phenological differences of the species and the seasonality of the water availability, which are important for competition between these species. Thus, our study can only provide limited evidence that global climate change is unlikely to facilitate the spread of *B. tectorum* dominance into those sagebrush-steppe communities with an undisturbed perennial bunchgrass component. However, the positive effects that elevated temperatures, reduced water availability, and elevated nutrients had on *B. tectorum’s* competitiveness, in addition to its positive response to CO_2_ when grown without interspecific competition, demonstrate the importance of limiting human-caused disturbance and maintaining intact native sagebrush-steppe communities.

## Electronic supplementary material

Below is the link to the electronic supplementary material.

Supplementary material 1 (PDF 12 kb)
