# Supplementary material for: Competition between cheatgrass and bluebunch wheatgrass is altered by temperature, resource availability, and atmospheric CO2 concentration
Source: Oecologia. 2017 Dec 22;186(3):855–68. doi: 10.1007/s00442-017-4046-6 (PMC5829107; doi:10.1007/s00442-017-4046-6)
Supplement: Supplementary file 1 — Supplementary material 1 (PDF 12 kb) [file 442_2017_4046_MOESM1_ESM.pdf]

| Treatment | Temperature | NPK | Water |
| --- | --- | --- | --- |
| 1 | + | + | + |
| 2 | + | + | - |
| 3 | + | - | + |
| 4 | + | - | - |
| 5 | - | + | + |
| 6 | - | + | - |
| 7 | - | - | + |
| 8 | - | - | - |

**Online Resource 1** Full factorial experimental design for experiment one across two temperature levels, ambient (-) and elevated (+), two nutrient levels, ambient (-) and elevated (+), and two water levels, ambient (+) and decreased (-). The eight treatment combinations were replicated twice in each of the five density treatments for both trials
